# Early prediction of survival after open surgical repair of ruptured abdominal aortic aneurysms

**DOI:** 10.1186/1471-2482-14-92

**Published:** 2014-11-18

**Authors:** Felix Krenzien, Ivan Matia, Georg Wiltberger, Hans-Michael Hau, Moritz Schmelzle, Sven Jonas, Udo X Kaisers, Peter T Fellmer

**Affiliations:** Department of Visceral, Transplantation, Thoracic and Vascular Surgery, University Hospital of Leipzig, Leipzig, Germany; Transplant Surgery Research Laboratory and Division of Transplant Surgery, Brigham and Women’s Hospital, Harvard Medical School, Boston, MA USA; Department of Anesthesiology and Intensive Care Medicine, University Hospital of Leipzig, Leipzig, Germany

**Keywords:** Abdominal, Aortic aneurysm, Aneurysm, Ruptured, Scoring Methods, Mortality, Critically Ill

## Abstract

**Background:**

Scoring models are widely established in the intensive care unit (ICU). However, the importance in patients with ruptured abdominal aortic aneurysm (RAAA) remains unclear. Our aim was to analyze scoring systems as predictors of survival in patients undergoing open surgical repair (OSR) for RAAA.

**Methods:**

This is a retrospective study in critically ill patients in a surgical ICU at a university hospital. Sixty-eight patients with RAAA were treated between February 2005 and June 2013. Serial measurements of Sequential Organ Failure Assessment score (SOFA), Simplified Acute Physiology Score II (SAPS II) and Simplified Therapeutic Intervention Scoring System-28 (TISS-28) were evaluated with respect to in-hospital mortality. Eleven patients had to be excluded from this study because 6 underwent endovascular repair and 5 died before they could be admitted to the ICU.

**Results:**

All patients underwent OSR. The initial, highest, and mean of SOFA and SAPS II scores correlated significant with in-hospital mortality. In contrast, TISS-28 was inferior and showed a smaller area under the receiver operating curve. The cut-off point for SOFA showed the best performance in terms of sensitivity and specificity. An initial SOFA score below 9 predicted an in-hospital mortality of 16.2% (95% CI, 4.3–28.1) and a score above 9 predicted an in-hospital mortality of 73.7% (95% CI, 53.8–93.5, p < 0.01). Trend analysis showed the largest effect on SAPS II. When the score increased or was unchanged within the first 48 h (score >45), the in-hospital mortality rate was 85.7% (95% CI, 67.4–100, p < 0.01) versus 31.6% (95% CI, 10.7–52.5, p = 0.01) when it decreased. On multiple regression analysis, only the mean of the SOFA score showed a significant predictive capacity with regards to mortality (odds ratio 1.77; 95% CI, 1.19–2.64; p < 0.01).

**Conclusion:**

SOFA and SAPS II scores were able to predict in-hospital mortality in RAAA within 48 h after OSR. According to cut-off points, an increase or decrease in SOFA and SAPS II scores improved sensitivity and specificity.

## Background

The mortality for ruptured abdominal aortic aneurysm (RAAA) remains high in the face of medical progress. Approximately 1% to 2% of all deaths in the western population are caused by RAAA [1, 2]. The therapeutic options include endovascular aneurysm repair (EVAR) and open surgical repair (OSR). Systematic reviews based on observational studies suggest survival benefits for endovascular treatment when compared with OSR [3–5]. Of note, no significant difference was found in recently published randomized controlled studies [6–8]. It remains unclear whether the controversial results are due to superior treatment or patient selection.

Interestingly, there are predictors of survival independent of the chosen treatment: Hemodynamic shock, loss of consciousness, sex and the anatomy of the aneurysm [6, 9–14]. Not only do preoperative factors predict mortality in RAAA, but postoperative condition might also have a clinical impact on survival. There are several outcome prediction models in the intensive care unit (ICU) environment. The Sequential Organ Failure Assessment score (SOFA) composed of scores from six organ systems determines the grade of organ dysfunction and failure [15]. The Simplified Therapeutic Intervention Scoring System-28 (TISS-28) is assessed according to therapeutic activities using 28 items [16]. The Simplified Acute Physiology Score II (SAPS II) is based on 12 physiological variables, age and type of admission [17]. These three models are widely used by ICUs to track patients and to predict clinical outcome [15, 18, 19].

Patients with RAAA who undergo OSR may die quickly within days or after weeks in the ICU. Early postoperative prediction of mortality in these patients is questionable but could lead to an adjustment of treatment according to outcome. Furthermore, these scoring models can be used to compare the performance of different departments or can be used to match cohorts according to their critical illness. To date the importance of the proposed scoring models in patients with RAAA is not fully clear and no competitive day by day analysis has yet been performed. The aim of the present study was to evaluate SOFA, SAPS II, and TISS-28 measurements as predictors of survival in a surgical ICU in patients with RAAA treated by OSR.

## Methods

### Setting

This retrospective study was conducted at the Division for Vascular Surgery, Department of Surgery, University Hospital Leipzig, Germany. The ICU provides 58 beds exclusively for surgical patients and is guided by the Department of Anesthesiology and Intensive Care Medicine. The study was reviewed and approved by the ethics committee of the University of Leipzig.

### Patients

Sixty-eight patients with RAAAs were treated between February 2005 and June 2013. Only those patients who underwent OSR and were treated in the ICU were included in this study. Hence, 11 patients had to be excluded because 6 of them underwent EVAR and 5 died before they could be admitted to the ICU. The medical records were reviewed retrospectively based on clinical characteristics and outcome. The rupture of the abdominal aorta was assured according the operative report. One-year follow up was carried out retrospectively and 9 patients (14.5%; 95% confidence interval, 5.8–23.3) were lost to follow up.

### Data collection

The data collection in the ICU was performed by a clinical information system (Copra System GmbH, Sasbachwalden, Germany). Medical work and care duties were captured in the electronic records along with automatically collected data from ventilators, vital signs and infusion systems. The SAPS II, SOFA and TISS-28 scores were assessed prospectively in our study. Every score was calculated on a daily basis at 6 a.m. in the morning. According to the standard documentation process, SAPS II and TISS-28 were also scored on the day of admission. Furthermore, the highest score during the stay in the ICU and the mean score were determined for each model. An ‘increase or no change’ was defined as any assessed score higher than or equal to the initial score within 48 h. A ‘decrease’ was determined as any score below the initial score within 48 h.

### Scoring models

The SOFA score (0–24) is based on six different organ systems: PaO_2_/FiO_2_ for respiratory failure; creatinine level or urine output for renal failure; bilirubin for liver failure; Glasgow Coma Scale (GAS) for neurological status; platelet count for coagulation; and mean arterial pressure or administration of vasopressors for cardiovascular system [15]. The SAPS II score (0–163) include 17 variables composed by 12 physiological variables, age, type of admission and three different underlying disease variables [17]. The TISS-28 score (1–78) derive from 28 therapeutic activities performed on the ICU subdivided into 7 groups: basic activities, ventilatory support, cardiovascular support, renal support, neurological support, metabolic support, and specific interventions [16].

### Statistical analyses

Statistical analysis was performed using SPSS (version 20; SPSS, Inc., Chicago, IL, USA). Continuous variables are presented as median and categorical values as percentages. The confidence interval (CI) was determined at 95%. The chi-square-test with Yates’ correction was applied to test univariate differences between dichotomous variables. Continuous variables between survivors and non-survivors were assumed to be non-normally distributed and were compared using a non-parametric Mann–Whitney U-test. To assess the discriminative power of the different scores to predict whether the patient will survive or die, the area under the receiver operating characteristic curve (ROC) was calculated. In addition, the Youden index *J* was defined to capture an optimal cut-off point for each score and point in time [20]:

$$J=\max\left\{\mathrm{sensitivity}+\mathrm{specificity}-1\right\}$$

This threshold represents the point with the highest sensitivity and specificity. Graphically, J is the maximum vertical distance between the ROC curve and 45-degree diagonal line. Furthermore a univariate analysis was carried out to evaluate the link between in-hospital mortality and the scoring model. A multiple logistic regression analysis was performed to evaluate a possible independent effect of significant factors detected in the univariate analysis. A selection of predictive variables was done by an automatic stepwise procedure in a forward–backward mode, and those with a significance <0.10 were entered into the multiple analysis. The correct classification rate (CCR) for the best model was reported. A P <0.05 was defined as significant.

## Results

The overall in-hospital mortality of patients with RAAA who underwent OSR was 41.9% (95% CI, 22–45.8) and the one-year mortality was 49.7% (95% CI, 29.6–54.3). In Table 1 are listed all baseline characteristics divided into survivor and non-survivor subsets. Neither group showed statistically significant differences with respect to diabetes mellitus, cardiovascular or pulmonary co-morbidities. The patients who died were significantly older, with a median age of 80.9 years (95% CI, 75.7–84.5).

**Table 1:** **Baseline characteristics of study population**

	Survivor (n =36)	Non-survivor (n =26)	***p***
Gender			
Male	86% (31/36)	73% (19/26)	0.2
Female	14% (5/36)	27% (7/26)	
Age at presentation	72.1	80.9	<0.01*
LOS (days)	17.7	4.8	<0.01*
ICU (days)	4.7	3.2	0.54*
Readmission on ICU (n)	11% (4/36)	8% (2/26)	0.65
Cardiovascular co-morbidity	92% (33/36)	81% (21/26)	0.21
Pulmonary co-morbidity	28% (10/36)	35% (9/26)	0.56
Diabetes mellitus	25% (9/36)	8% (2/26)	0.08
BMI (kg/m^2^)	26	26.4	0.85*

Baseline characteristics of the patients are subdivided into survivors and non-survivors.

Statistical significance was assessed by the chi-square-test with Yates’ correction and the Mann–Whitney U-test*.

LOS = length of stay; ICU = intensive care unit; BMI = body-mass index.

### SOFA

**Figure 1:**
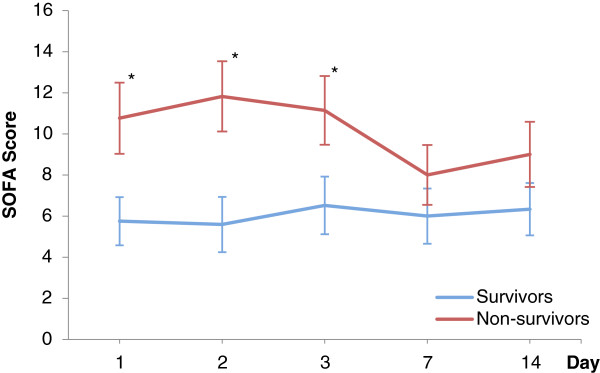
**SOFA score for survivors and non-survivors.** The SOFA score is plotted respectively for 57 patients after OSR of ruptured abdominal aortic aneurysm. For each time point 95% CI is shown. Both subgroups were compared by using the Mann–Whitney U-test (*P <0.05).

**Figure 2:**
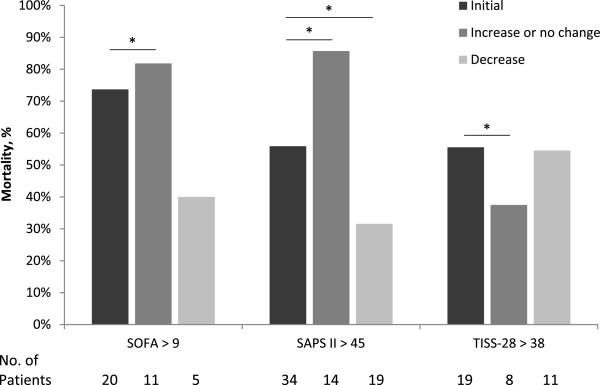
**Trend analysis within 48 h after surgery of SOFA, SAPS II and TISS-28.** The in-hospital mortality rates are graphed for the initial cut-off point calculation of each scoring model and the following trend within 48 h (increase or no change; decrease). Chi-square-test with Yates’ correction was performed by comparing the initial score versus ‘increase or no change’ and ‘decrease’ (*P <0.05).

After admission to the ICU, the SOFA scores for survivors and non-survivors were 5.8 (95% CI, 4.6–6.9) and 10.8 (95% CI, 9–12.5, p < 0.01), respectively (see Figure 1). The calculation of the ROC curves and the corresponding cut-off point with sensitivity and specificity is depicted in Table 2. The mean SOFA score showed the highest area under the ROC curve (0.92; 95% CI, 0.81 - 0.97). To enable early prediction of in-hospital mortality, the optimal cut-off value was determined. The in-hospital mortality rate for an initial SOFA score of up to 9 was 16.2% (95% CI, 4.3–28.1) and the in-hospital mortality rate for a SOFA score of above 9 was 73.7% (95% CI, 53.8–93.5, p < 0.01). To improve the sensitivity and specificity of cut-off points, trends of the scoring system were analyzed (see Figure 2). When the SOFA score (initially >9) did not change or increased within 48 h, the in-hospital mortality rose to 81.8% (95% CI, 59–100, p = 0.03) and was 40% (95% CI, 0–82.9, p = 0.31) when the score decreased.

**Table 2:** **Comparisons of the areas under the ROC curves for prediction of mortality**

	Cut-off point	Sensitivity/Specificity	AUC	p
SOFA				
24 h	> 9	71.4/86.1	0.79 (95% CI, 0.67 - 0,89)	< 0.01
48 h	> 9	76.5/85.3	0.83 (95% CI, 0.70 - 0.92)	< 0.01
72 h	> 10	78.6/77.8	0.79 (95% CI, 0.63 - 0.90)	< 0.01
Mean	> 7.25	85.7/85.7	0.92 (95% CI, 0.81 - 0.97)	< 0.01
Max	> 9	95.2/71.4	0.86 (95% CI, 0.75 - 0.94)	< 0.01
SAPS II				
Initial	> 45	90/58.3	0.76 (95% CI, 0.62 - 0.86)	< 0.01
24 h	> 40	95/60	0.85 (95% CI, 0.73 - 0.93)	< 0.01
48 h	> 37	93.7/56.2	0.80 (95% CI, 0.66 - 0.90)	< 0.01
72 h	> 43	85.7/65.2	0.73 (95% CI, 0.55 - 0.86)	0.01
Mean	> 43.4	95.2/80.6	0.91 (95% CI, 0.80 - 0.97)	< 0.01
Max	> 54	95.2/69.4	0.88 (95% CI, 0.76 - 0.95)	< 0.01
TISS-28				
Initial	> 38	52.6/73.3	0.58 (95% CI, 0.43 - 0.72)	0.35
24 h	> 34	73.7/62.9	0.71 (95% CI, 0.57 - 0.82)	< 0.01
48 h	> 32	80/60	0.73 (95% CI, 0.58 - 0.85)	< 0.01
72 h	> 32	92.3/52.2	0.77 (95% CI, 0.61 - 0.90)	< 0.01
Mean	> 36.25	70/91.4	0.86 (95% CI, 0.74 - 0.94)	< 0.01
Max	> 47	61.9/82.9	0.74 (95% CI, 0.61 - 0.85)	< 0.01

Calculation of ROC curves for the different models. The cut-off point is the optimal threshold to distinguish between survivors and non-survivors. The sensitivity and specificity correspond to the cut-off point. Mean Score was calculated as the average of every assessed score.

AUC = area under the curve; SOFA = Sequential Organ Failure Assessment; SAPS II = Simplified Acute Physiology Score II; TISS-28 = Simplified Therapeutic Intervention Scoring System-28.

### SAPS II

**Figure 3:**
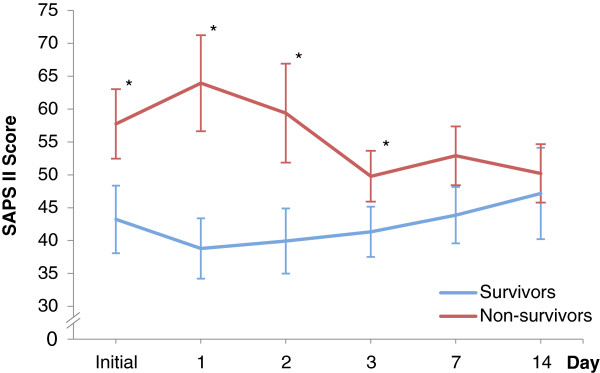
**SAPS II score for survivors and non-survivors.** The SAPS II score is plotted respectively for 57 patients after OSR of ruptured abdominal aortic aneurysm. For each time point the 95% CI is shown. Both subgroups were compared by using the Mann–Whitney U-test (*P <0.05).

The SAPS II score was calculated upon admission. The mean score was 43.2 (95% CI, 38.1–48.4) for survivors and 57.8 (95% CI, 52.5–63.1, p < 0.01) for non-survivors (see Figure 3). The best predictive model for SAPS II was the mean value that had an area under the ROC curve of 0.91 (95% CI, 0.80 - 0.97). The cut-off point for the initial SAPS II score was >45. A score ≤45 predicted a mortality of 8.6% (95% CI, 0–20.4) and a score >45 predicted an in-hospital mortality rate of 55.9% (95% CI, 39.2–72.6, p < 0.01). When the score increased or did not change within 48 h and the initial value scored >45, the in-hospital mortality rate was 85.7% (95% CI, 67.4–100, p < 0.01). In contrast, the in-hospital mortality rate was 31.6% (95% CI, 10.7–52.5, p = 0.01) when the score decreased.

### TISS-28

**Figure 4:**
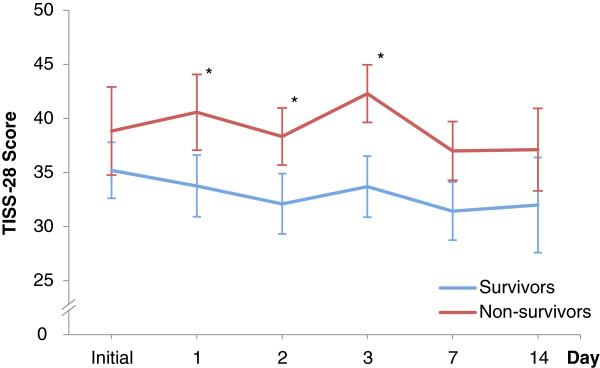
**TISS-28 score for survivors and non-survivors.** The TISS-28 score is plotted respectively for 55 patients after OSR of ruptured abdominal aortic aneurysm. For each time point the 95% CI is shown. Both subgroups were compared by using the Mann–Whitney U-test (*P <0.05).

The TISS-28 was scored on the day of admission. For survivors and non-survivors the scores were 35.2 (95% CI, 32.6–37.8) and 38.8 (95% CI, 34.8–42.9, p = 0.33), respectively (see Figure 4). The largest area under the ROC curve of 0.86 (95% CI, 0.74 - 0.94) was evaluated for the mean value. In consideration of the cut-off value, the in-hospital mortality of the initial TISS-28 score was 29% (95% CI, 13.1–45.0) if the value was ≤38 and it was 55.5% (95% CI, 32.6–78.5, p = 0.07) if the value was >38. For initial scores >38, the in-hospital mortality rate was 37.5% (95% CI, 32.6–78.5, p < 0.01) if there was an increase or no change and the in-hospital mortality rate was 54.5% (95% CI, 25.1–84, p = 0.07) if it decreased.

Significant scores according univariate analysis, including mean and maximum values as well as several time points, were taken into a multiple analysis. However, only the mean SOFA score showed a simultaneous independent effect with regards to in-hospital mortality (odds ratio 1.77; 95% CI, 1.19–2.64; p < 0.01). The CCR for this model was 80%.

## Discussion

The present study evaluated scoring models in patients with RAAA after OSR and demonstrated their feasibility in the environment of a surgical ICU. SOFA, SAPS II and TISS-28 scores were able to predict mortality within 48 h after surgery even though there were differences in terms of sensitivity, specificity and trend analysis. To the best of our knowledge, this is the first competitive day by day analysis of the presented scoring systems for RAAA including trend analysis. Moreover, for the first time, TISS-28 was evaluated in patients with RAAA.

The tested scores were developed with assistance of statistical modeling technique [15–17]. In contrast, older scores like APACHE or APACHE II were built by a subjective method as experts selected and weight variables from a panel to compose these scores [21, 22]. Nevertheless, especially APACHE II and Multiple organ dysfunction score showed its applicability in RAAA, but with variance in discrimination to predict outcome [23–26]. The comparison between these studies is difficult due different designs and clinical settings. Thus, local customization and validation of scores is even more important and can improve discrimination power [27, 28]. Interestingly, same parameters are considered in different scores. For instance, parameters of APACHE II like gas exchange and acid–base balance (pH, HCO3, PaO2, FiO2, creatinine, potassium, age, systolic blood pressure) are assessed by SAPS II. The presented scores were validated mainly in surgical patients [18, 27, 29], but their applicability to the vascular field are unclear. Therefore, the evaluation in RAAA is essential because of individual differences from score to score.

The proposed scoring models are based on postoperative parameters. They can not only be used to predict outcome, even critical illness can be tracked day by day. Hence, they are in sharp contrast to GAS, Edinburgh Rupture Aneurysm Score (ERAS) or Hardman Index [13, 30, 31]. These scores are composed by preoperative parameters and can be calculated in the emergency department to determine the likelihood whether a patient will survive or die. Interestingly, these scores were developed exclusively in patients with RAAA.

The discriminative power to distinguish between survivors and non-survivors according the ROC calculation after surgery was highest for mean SOFA followed by mean SAPS II and mean TISS-28. The highest values were found in means, what can be explained by a higher numbers of values, which were considered in the statistical analysis. Previous studies confirm the results for SOFA scores in critically ill patients (AUC range: 0.69 to 0.92) [29, 32]. The scoring systems appear to have a trend towards outcome within the first 48 h. In consideration of the serial measurements of Figures 1, 3 and 4, the values increased for survivors and decreased for non-survivors. A trend analysis was carried out to improve the discriminative power of cut-off values in scoring models and SAPS II showed the best performance. SAPS II (cut-off value >45) had a significant increase of almost 30% mortality when the score increased or did not change and a significantly lower mortality of 25% when the score decreased. In the trend analysis of SOFA, only a slightly higher mortality (8%) was found. This might be affected by the assessment at 6 a.m. according to our documentation policy. Potentially dynamic changes of SOFA score may arise directly after surgery, which would be consistent with recently published studies [32, 33]. In contrast, the threshold of TISS-28 was able to predict mortality (29% vs. 55%) but changes in that value could not improve sensitivity and specificity. Overall, TISS-28 was inferior to SOFA and SAPS in terms of ROC calculation and predicting outcome.

Since 2005, all three investigated models were assessed semi-automatically in our clinical information system. The widespread availability of electronic devices in the ICU environment led to the recording of vital signs, medication, care duties and medical work. Patients with RAAA represent a high-risk group for complications and mortality. Although the implementation of scoring systems need additional effort, it may allow inter-individual decision-making and may provide information for relatives.

New generations of ICU scoring systems are promising, e.g. APACHE III or SAPS III, and of high interest for future studies [27, 28]. However, the present study assessed the evaluated scores prospectively during the clinical routine and a consideration of additional new scores would include a major confounder in terms of a retrospective calculation prone to missing values.

There are limitations in the present analysis. The most important are the retrospective review of the medical records and the underpowered number of patients. Due to the mortality rate over time, the numbers of patients and measurement drops in relation to time. Thus, an analysis of a certain time point after one week might not be substantial. The reported in-hospital mortality of 41.7% is consistent with previous reports. Reimerink et al. reports a mortality rate of 49% (45% to 55%) in a meta-analysis [34]. Patient co-morbidities of both groups did not differ significantly. The median age of the present cohort was 75.9 (IQR; 64.6 – 80.7), what is consistent to large epidemiological studies [34]. The survivor group was younger than the non-survivor group (72.1 years vs. 80.9 years). An advanced age is a negative predictor for survival [35], but age is not considered in the SOFA and TISS-28. Strikingly, these scores were able to predict patients, who will die and will be older. In a recent study, SAPS II was able to predict mortality in patients >90 years [36]. Of note, the tested scores are applicable in the elderly.

Patients treated by EVAR were excluded from this study to test the scores exclusively in patients’ who underwent open surgery, as a single treatment option. Clearly, patients who underwent surgery or endovascular repair have major differences in postoperative morbidities and physiological changes [7, 37, 38]. Moreover, patients who undergo EVAR are selected and biased. EVAR suitability is determined by the anatomic configuration of the aortic neck and iliac arteries, while OSR is not limited by the aneurysm morphology. Thus, patients, who are not suitable for EVAR, usually undergo surgery, as reflected in observational studies [39, 40]. Therefore, these patients were excluded from our analysis.

Serial measurements of scoring models have a big impact on the assessment of critically ill patients. Most ICU’s are directed by anesthesiologist and primary sections like vascular surgery are losing influence in terms of the handling and assessment of these scores. Therefore, the evaluation of widely used scores in vascular surgical patients is indispensable to ensure an objective assessment of vascular patients in the ICU and to avoid interpreting scores on the basis of a heterogeneous patient cohort.

## Conclusion

The present study suggests SOFA and SAPS II scores for early prediction of in-hospital mortality in RAAA. The score trend within 48 h of SOFA and SAPS II improves sensitivity and specificity. Hence, mortality in RAAA is not determined solely by surgery as even perioperative management and factors influence the clinical outcome. The presented scoring models are suitable to track patients as well as performance of different departments and can be used to match patient groups according to their risk.

## Abbreviations

RAAA: Ruptured abdominal aortic aneurysm
OSR: Open surgical repair
EVAR: Endovascular aneurysm repair
SOFA: Sequential organ failure assessment score
SAPS II: Simplified acute physiology score II
TISS-28: Simplified therapeutic intervention scoring system-28
ICU: Intensive care unit
GAS: Glasgow coma scale
CCR: Correct classification rate
ROC: Receiver operating characteristic
CI: Confidence interval
AUC: Area under the curve.
